# TRP Channels as Therapeutic Targets in Diabetes and Obesity

**DOI:** 10.3390/ph9030050

**Published:** 2016-08-17

**Authors:** Andrea Zsombok, Andrei V. Derbenev

**Affiliations:** 1 Department of Physiology, School of Medicine, Tulane University, New Orleans, LA 70112, USA; aderben@tulane.edu; 2 Department of Medicine, Endocrinology Section, School of Medicine, Tulane University, New Orleans, LA 70112, USA

**Keywords:** TRPV1, TRPM, TRPA1, obesity, diabetes mellitus, metabolism, glucose homeostasis

## Abstract

During the last three to four decades the prevalence of obesity and diabetes mellitus has greatly increased worldwide, including in the United States. Both the short- and long-term forecasts predict serious consequences for the near future, and encourage the development of solutions for the prevention and management of obesity and diabetes mellitus. Transient receptor potential (TRP) channels were identified in tissues and organs important for the control of whole body metabolism. A variety of TRP channels has been shown to play a role in the regulation of hormone release, energy expenditure, pancreatic function, and neurotransmitter release in control, obese and/or diabetic conditions. Moreover, dietary supplementation of natural ligands of TRP channels has been shown to have potential beneficial effects in obese and diabetic conditions. These findings raised the interest and likelihood for potential drug development. In this mini-review, we discuss possibilities for better management of obesity and diabetes mellitus based on TRP-dependent mechanisms.

## 1. Introduction

The World Health Organization estimates that obesity doubled between 1980 and 2014, with 39% of adults being overweight and 13% being obese in 2014. In the United States the statistics are similarly alarming, more than one-third of the adults are obese and 17% of youth are also obese [1,2]. The consequences of obesity are severe including higher mortality rate, hypertension, dyslipidemia, heart disease, stroke, type 2 diabetes mellitus (T2DM), and many more.

Among the various pathophysiological conditions, T2DM is very prevalent and affects approximately 10% of the population worldwide. Lifestyle changes including weight loss can result in better glucose management; however, lifestyle changes require disciplined behavior and frequent monitoring. Unfortunately, patients often give up before reaching their goals or detectable results. In recent years, bariatric surgery has emerged as an effective treatment for improvement or remission of T2DM; however, due to the invasiveness of the procedure and potential surgical complications, bariatric surgery is not the most feasible solution. The most common treatments are still pharmacological interventions. Current pharmacological treatments mainly target the end organs by increasing insulin secretion from pancreatic beta cells (e.g., sulfonylureas), increasing tissue sensitivity to insulin (e.g., metformin), modulating the glucagon-like peptide 1 (GLP-1) system, preventing glucose reabsorption in the kidneys (SGLT2 inhibitors) or administering insulin. Despite the existing treatments, new therapeutic interventions are in great demand, therefore, every new idea, pathway, receptor, or compound (natural or synthetic) which has the potential of developing a new class of drugs for weight and/or glucose management receives attention.

Members of the transient receptor potential (TRP) family have been identified as key contributors in many physiological and pathophysiological conditions [3]. TRP channels were identified in numerous metabolically important tissues. Members of the canonical (TRPC), melastatin (TRPM), ankyrin (TRPA1), and vanilloid (TRPV) subfamilies were found to be expressed in the pancreas [3,4], liver, gastrointestinal tract [5], skeletal muscle, kidney [6,7,8], adipose tissue [9,10,11,12], heart [13,14,15], vasculature [16,17,18], and nervous system [19,20], including autonomic centers of the brain [21,22]. Despite that TRP channels and their natural ligands received considerable attention in the field of obesity and diabetes [4,23,24,25,26,27,28], their role in many of the metabolic processes are still debated and under investigation. This mini-review summarizes findings demonstrating functional roles of TRP channels in obese and diabetic conditions.

## 2. TRPV1 for the Prevention and Treatment of Obesity and Diabetes Mellitus

TRPV1 is one of the most studied TRP channels even in the context of obesity and diabetes mellitus. TRPV1, the first described member of the vanilloid subfamily, was cloned in 1997 [29]. It is a nonselective cation channel with permeability to Ca^2+^. In the past decades TRPV1 was extensively researched and investigated as potential drug target for a variety of applications [23,30,31]. Numerous findings related to TRPV1 including its structure, species-related differences, topology, agonists, antagonists, cellular mechanisms, and pharmacological applicability can be found elsewhere including in a review by Nilius and Szallasi [23]. TRPV1 is activated by a variety of exogenous and endogenous ligands [23,32]. Among the well-known exogenous ligands, capsaicin—the pungent ingredient of hot peppers—is investigated the most for its beneficial effects on body weight and metabolism. Capsaicin-binding site of TRPV1 is intracellular [33]. Activation of TRPV1 results in influx of cations and leads to depolarization of the cell [34,35]. Capsaicin specifically binds to TRPV1; however, we have to keep in mind that capsaicin is able to cause disruption in the organization of membranes due to its localization in the lipid bilayer [36,37,38]. This might have important implications for delivery of drug molecules and may result in a TRPV1-independent effect.

Among the numerous physiological functions, TRPV1 has been proposed to have functional roles in a variety of metabolically important tissues including the pancreas [39,40], gastrointestinal tract [41], adipose tissue [9,12], and nervous system [21,42]. In this section, based on human and animal model studies, we provide a brief overview of TRPV1’s potential for improvement of obese and diabetic conditions.

### 2.1. Dietary Interventions and Possible Mechanisms in Human and Animal Model Studies

Ingredients of spices, such as capsaicin in red peppers, are well-known for a variety of effects. Recently, Nilius and Appendino published a detailed overview about the beneficial science of spices, focusing on TRPs [28], while in this section we summarize dietary interventions and potential underlying mechanisms related to TRPV1.

Previous studies in male human subjects examined the effects of red pepper diet and demonstrated a trend for increased energy expenditure (EE), significantly higher carbohydrate oxidation, and lower lipid oxidation [43]. This increased EE immediately after a red pepper containing meal was proposed to be due to beta-adrenergic stimulation [43]. Increased carbohydrate oxidation and elevated epinephrine and norepinephrine levels following dietary red pepper ingestion were confirmed in men by another study [44].

In Japanese female subjects, diet-induced thermogenesis and lipid oxidation was higher following red pepper diet compared to the control diet [45]. On the other hand, Matsumoto and co-workers found that diet-induced thermogenesis was increased in the age- and height-matched lean control group; however, there was no thermogenic response detected in the obese group [46]. The study demonstrated that despite the identical resting sympathovagal activities, reduced sympathetic responsiveness to the capsaicin diet exists in obese females. This reduced sympathetic responsiveness likely leads to impairment of the diet-induced thermogenic response and could be an important factor in the development of obesity in female subjects [46].

A crossover study with male and female participants also aimed to determine the effect of a capsaicin-containing lunch on EE and hormone levels [47]. This study revealed increased GLP-1 levels and a decreasing trend in ghrelin levels; however, no effect on satiety, EE, and peptide YY levels were observed [47]. The negative finding on satiety was in disagreement with a previous study from the same group, in which significantly higher feelings of satiety were seen after capsaicin supplementation over two days compared to placebo [48]. The authors suggested that the satiety feeling observed after capsaicin need to be built up, and the postprandial state may mask the effects of capsaicin in general [47,48].

Many of the studies demonstrating beneficial findings of capsaicin investigated a single meal in a small group of lean subjects [45,49], while other studies focused on determining the effects of weeks long chili diet. The effect of daily ingestion of chili pepper for four weeks was investigated on the resistance of serum lipoproteins to oxidation in healthy men and women [50]. The rate of oxidation was lower following the chili pepper diet compared to the bland diet, suggesting that regular consumption of the chili diet for an extended period inhibits oxidation of serum lipoproteins; however, in addition to capsaicin, other ingredients of chili pepper can contribute to these findings [50].

On the other hand, the same group revealed no obvious beneficial or harmful effects on metabolic and vascular parameters, but suggested that four week long chili consumption may reduce resting heart rate and increase effective myocardial perfusion pressure time in men [51].

A crossover intervention study by the same group used two dietary periods of four weeks each and measured EE, serum insulin, C-peptide, and glucose levels following a bland meal after bland diet, a chili meal after bland diet, and a chili meal after chili-containing diet and suggested that regular consumption of chili may attenuate postprandial hyperinsulinemia [52].

Lejeune and co-workers revealed no difference in body weight maintenance after body weight loss following capsaicin consumption for multiple weeks compared to the placebo group [53]. However, substrate oxidation was affected by capsaicin, which was consistent with the observation of a short-term study by Yoshioka [54].

In general, as demonstrated by the abovementioned examples, in human subjects there are controversial findings regarding capsaicin containing diets, and clearly more detailed studies are necessary to make a conclusion about the dietary effects of capsaicin and TRPV1-activating supplements. We also have to note that many variables including age, metabolic status, postprandial state, sex, the diet itself (e.g., chili pepper vs. capsaicin), and many more can profoundly influence or even mask the metabolic effects in the above-discussed human studies.

Despite that the animal model studies are more mechanistic, they also revealed controversial findings about the metabolic effects of TRPV1 activation. Major dietary and topical interventions in obese and diabetic conditions were recently reviewed in more detail [4,26]. Briefly, it has been reported that TRPV1 activation with capsaicin prevented adipogenesis and obesity [9]. The study verified TRPV1 expression in 3T3-L1-preadipocytes and visceral adipose tissue from mice and humans. The findings revealed that capsaicin dose-dependently increased intracellular calcium in 3T3-L1-preadipocytes, which indicates inhibition of preadipocytes’ differentiation. The capsaicin-dependent calcium increase was significantly lower in mature adipocytes compared to preadipocytes, which was consistent with the observed TRPV1 downregulation during adipogenesis [9]. This study also revealed that both db/db and ob/ob mice have lower TRPV1 expression in their visceral adipose tissue compared with the lean controls. Indeed, lower TRPV1 expression was determined in visceral and subcutaneous adipose tissue of obese men compared to lean men [9]. These observations suggest that downregulation of TRPV1 in visceral adipose tissue is a common finding both in animal models of obesity and diabetes, and in human subjects.

Dietary capsaicin (0.01%) did not affect the body weight of mice on normal chow diet; however, dietary supplementation of capsaicin to high fat diet (HFD) treated mice prevented obesity, which was not observed in TRPV1 knockout mice [9]. The prevention of obesity was associated with small adipocyte size and increased TRPV1 expression. These findings suggest that activation of TRPV1 triggers calcium influx, and leads to prevention of adipogenesis and TRPV1 downregulation that may result in attenuation of obesity in mice kept on HFD.

A more recent study evaluated the effect of dietary capsaicin (0.01%) on browning of white adipose tissue (WAT) [55]. Capsaicin diet prevented the weight gain of wild type mice on HFD, while this effect was not observed in TRPV1 knockout mice. HFD downregulated the expression of TRPV1 in epididymal and subcutaneous fat pads, and the capsaicin diet prevented this downregulation and caused browning of WAT. The prevention of obesity was associated with increased uncoupling protein 1 (UCP1) expression levels, and increased sirtuin-1 expression and activity. These findings were due to TRPV1-dependent increase of Ca^2+^ and phosphorylation of CaMKII and AMPK. TRPV1 activation elevated metabolic and ambulatory activities and induced browning of WAT likely via peroxisome proliferator-activated receptor gamma (PPAR-γ)/PRDM-16 interaction [55]. These results suggest that TRPV1 activation could be used to promote browning of WAT and thus decrease obesity (Table 1).

We have to mention that in addition to TRPV1, TRPV3 and TRPV4 have been shown to have functional roles in adipocytes. Recently, higher expression of TRPV3 was found in adipocytes when compared to other TRPVs. Activation of TRPV3 was shown to prevent lipid accumulation and adipogenesis by inhibiting the phosphorylation of insulin receptor substrate 1 and the expression of PPAR-γ [77]. Moreover, HFD-treated, db/db, and ob/ob mice had reduced TRPV3 expression in their visceral adipose tissue, whereas chronic treatment with TRPV3 agonists prevented adipogenesis and weight gain in HFD-treated mice [77].

In contrast, Ye and co-workers found that TRPV1, TRPV2, and TRPV4 mRNA are expressed in 3TT-F442A adipocytes, but their study showed a lack of TRPV3 mRNA. TRPV4 was expressed at the highest level [78], which confirmed previous observations revealing TRPV4 expression in adipose tissue [79]. Furthermore, Ye’s paper determined that TRPV4 is a negative regulator of oxidative metabolism and it controls proinflammatory gene programming. In TRPV4 knockout mice the subcutaneous adipose tissue showed higher UCP1 mRNA and protein expression levels compared to controls. Elevated EE was found in the TRPV4 knockout mice, which was associated with the elevated thermogenic gene programming. In addition, TRPV4 knockout mice were protected from diet-induced obesity and insulin resistance [78]. Taken together, strong evidence suggests that TRPVs including TRPV1, TRPV3, and TRPV4 likely have important functional roles in adipocytes.

The effect of dietary hot pepper (1%) was determined to be beneficial to attenuate diet-induced obesity in rabbits [80]. TRPV1 mRNA was detected in various tissues of the rabbit with high similarity to human TRPV1. Rabbits fed with dietary hot pepper consumed similar amount of food; however, the hot pepper fed group gained less weight compared to the control group. This was associated with decreased adipose tissue and ratio of adipose tissue to body weight [80]. On the other hand, previous studies found that rabbits are insensitive to capsaicin and do not have resiniferatoxin binding sites [81]. It was later found that two amino acid substitutions in rabbit TRPV1 render the protein >100-fold less sensitive to vanilloids and RTX [82]. Therefore, it is debatable that the beneficial effects of dietary hot pepper observed by Yu [80] are mediated by capsaicin-dependent TRPV1 activation.

Male C57Bl/6 mice were kept on HFD for ten weeks and then received capsaicin (0.015%) supplement [70]. Dietary capsaicin resulted in lower fasting glucose, insulin, and leptin levels. In addition, it also attenuated the impairment of glucose tolerance, likely by reducing inflammatory responses and increasing fatty acid oxidation [70]. In another study by the same group, genetically obese diabetic mice (KKAy) were subjected to an HFD for two weeks then received capsaicin [72]. Dietary capsaicin reduced fasting glucose, insulin, and triglyceride levels, while adiponectin levels and its receptor expression were increased and inflammatory gene expression was decreased, resulting in reduced metabolic dysregulation.

In Swiss albino mice the effect of three month long treatment with capsaicin (2 mg/kg) plus HFD were compared with control diet and HFD [71]. The results demonstrated that capsaicin supplementation to HFD modulated hypothalamic satiety genes, altered gut microbial composition, induced browning of subcutaneous WAT, and increased thermogenesis in brown adipose tissue (BAT). Capsaicin-treated mice on HFD gained less weight than mice on HFD only. Fasting glucose levels were not different among the groups, whereas leptin and TNFα levels were decreased in the capsaicin-treated mice compared with the HFD group [71]. Hypothalamic expression of TRPV1 was downregulated in HDF mice and capsaicin treatment increased TRPV1 expression. Anorectic genes including peptide YY, brain-derived neurotrophic factor (BDNF) and CART prepropeptide were enhanced in capsaicin-treated HDF mice compared with HFD mice, while expression of orexigenic genes were reduced following capsaicin in the diet, with the exception of melanin-concentrating hormone receptor 1, hypocretin (orexin), and neuropeptide Y (NPY) [71]. The authors hypothesized that capsaicin activates vagal TRPV1 or afferent nerves in the gastrointestinal tract and thus influences hypothalamic TRPV1 and other genes’ expression.

Furthermore, TRPV1 is also known to modulate insulin secretion and pancreatic function, including development of islet inflammation and type 1 diabetes mellitus. These findings were described and reviewed earlier in detail [23,24,25,26,39,83].

### 2.2. Behind the Scene: Activating or Antagonizing TRPV1 for Treating Obesity and Diabetes Mellitus

Despite that various studies investigated TRPV1 activation, it is not clear whether activation or inhibition would be a better approach for the modulation of metabolism. Capsaicin-desensitized rats were protected from obesity despite atrophied BAT [11,84,85]. The body weight of capsaicin-desensitized rats was significantly lower 14 and 32 weeks after treatment. The lower body weight was associated with reduced food intake, smaller epididymal and retroperitoneal WAT depots, smaller interscapular BAT, decreased total protein, UCP, and cytochrome oxidase in BAT; however, the resting metabolic rate and colonic temperature of the groups were not different [11].

On the other hand, adult male rats, which were neonatally treated with capsaicin, did not differ in body weight, basal plasma leptin, or fasting leptin, insulin, adiponectin, and corticosterone levels [86]. Glucose levels following intravenous glucose tolerance tests were similar in the capsaicin-treated and vehicle-treated rats [86]. However, the capsaicin-treated rats displayed reduced plasma insulin and corticosterone responses, indicating increased insulin sensitivity and lower plasma corticosterone levels [86]. The increased insulin sensitivity was also supported by euglycemic hyperinsulinemic clamp studies [87]. Koopmans and co-workers suggested that adult rats with neonatal capsaicin treatment exhibit decreased corticosterone levels, which could contribute to amplified insulin action during hyperglycemia [86].

Studies using TRPV1 knockout mice also revealed conflicting results. On one hand, TRPV1 knockout mice gained less weight following HFD compared with wild-type mice [88]. In this study wild-type and TRPV1 knockout mice had similar energy intake, but TRPV1 knockout mice had greater thermogenic capacity. In contrast, in a more recent study TRPV1 knockout mice kept on HFD became more obese than the wild-type mice kept on the same HFD [89]. TRPV1 knockout mice on HFD were significantly heavier than the wild-type mice after one month of HFD, which was mainly due to increased whole-body fat mass. Indirect calorimetry demonstrated increased food intake of TRPV1 knockout mice on day 3. Decreased physical activity was observed during the night-cycle, which was consistent with reduced night-cycle energy expenditure rates in TRPV1 knockout mice [89]. TRPV1 knockout mice also developed more severe insulin and leptin resistance and they exhibited dysfunctional hypothalamic leptin signaling.

Marshall and co-workers investigated metabolic and cardiovascular parameters and found that wild-type and TRPV1 knockout mice gain a similar amount of weight on HFD [27]. In wild-type mice, HFD was associated with increased mean arterial pressure, which was not observed in TRPV1 knockout mice. Furthermore, parameters of vascular hypertrophy showed an increase in wild-type mice on HFD compared with TRPV1 knockout mice on HFD and mice on normal chow [27]. In the HFD-treated groups, baseline glucose levels showed an increasing trend, and impaired glucose tolerance was observed. Interestingly, glucose levels normalized faster in TRPV1 knockout mice, which was indicated by the area under the curve. Interleukin 10 and 1β levels were significantly elevated in wild-type, but not in TRPV1 knockout mice [27]. Based on their findings the authors suggested that TRPV1 deletion may be protective against obesity-induced hypertension, and that TRPV1 may contribute to the development of cardiometabolic disturbances. The role of TRP channels in the cardiovascular system can be found elsewhere [90].

Promising findings have been identified with TRPV1 antagonists. Since agonists can prevent TRPV1 signaling by desensitizing the receptor [17], TRPV1 antagonists may influence TRPV1 signaling by antagonizing the receptor, and thus improve obese and/or diabetic conditions. BCTC *N*-(4-tertiarybutylphenyl)-4-(3-chloropyridin-2-yl)tetrahydropyrazine-1(2*H*)-carbox-amide, a TRPV1 antagonist has been shown to inhibit TRPV1 signaling in cultured cells and was found to decrease inflammation and neuropathic pain in vivo [91,92]. Tanaka and co-workers investigated the effect of BCTC on whole-body glucose and lipid metabolism in ob/ob mice and compared the effect of BCTC with pioglitazone, an insulin sensitizer [56]. The insulin-resistant, hyperinsulinemic ob/ob mice were treated with BCTC or pioglitazone twice a day for four weeks. Plasma glucose levels were decreased with a high dose BCTC (100 mg/kg) and the decrease was similar to pioglitazone. Plasma insulin levels showed a dose-dependent decreasing trend following BCTC treatment, but significance was observed only in the pioglitazone group [56]. Plasma triglyceride levels were significantly lower both in the BCTC and pioglitazone-treated mice, which was accompanied with a decreasing trend in calcitonin gene-related peptide. The oral glucose tolerance test indicated that BCTC increased insulin secretion, but likely in a different way than pioglitazone, even though glucose tolerance was improved by both drugs [56]. The authors also reported that BCTC did not alter insulin or glucose levels in normoglycemic control mice, indicating that inhibiting TRPV1 could be more important during the diabetic condition [56]. In summary, BCTC was shown to improve insulin resistance, which may be due to inhibiting TRPV1 in adipocytes and skeletal muscle. On the other hand, increased insulin secretion was associated with BCTC in diabetic ob/ob mice. These findings suggest that BCTC may have a dual effect by improving insulin resistance and enhancing insulin secretion in ob/ob mice. We also have to note that BCTC is an antagonist of TRPM8 and we cannot exclude a possible effect and/or interaction with TRPM8 channels [93].

More recently, at the European Association for the Study of Diabetes 2015, a novel TRPV1 antagonist, AZV1 by Astra Zeneca, was shown to improve insulin sensitivity in ob/ob mice [57]. Mice were treated daily with the TRPV1 antagonist AZV1 or vehicle for eight days. Body weight of TRPV1 antagonist-treated mice was not different compared with vehicle-treated mice; however, the glucose control of ob/ob mice was improved. Glucose levels, homeostatic model assessment and fructosamine levels of the TRPV1 antagonist-treated mice were significantly decreased compared with vehicle-treated mice. Moreover, increased liver weight was observed in the TRPV1 antagonist treated mice [57]. The data demonstrated that AZV1 was well-tolerated without effects on food intake or body weight, but it exerted an antidiabetic effect, which may be due to improved insulin sensitivity. Findings from these two studies suggest that TRPV1 antagonism could be useful for the treatment of type 2 diabetes mellitus; however, we have to note that these antagonist studies did not investigate potential temperature changes.

Taken together, the beneficial effects of TRPV1 could be valuable; however, there are numerous unanswered questions about the mechanisms underlying the findings. These include sensitization vs. desensitization, important cell types/tissues/pathways underlying the effects (e.g., pancreatic beta cells vs. sensory nerves), or the contribution of the sympathetic nervous system.

## 3. Role of TRPM Channels in Metabolism

Members of the TRPM family have highly variable permeability to Ca^2+^ and they lack the *N*-terminal ankyrin repeats [23,94]. TRPM channels including TRPM2, TRPM3, TRPM4, and TRPM5 were identified as candidates to play a role in the regulation of metabolism [4].

Comprehensive metabolic studies were conducted to determine insulin sensitivity of TRPM2 knockout mice [95]. TRPM2 knockout mice were more insulin-sensitive due to increased glucose metabolism in the heart. TRPM2 knockout mice exerted increased EE and elevated expression levels of metabolic genes including peroxisome proliferator-activated receptor alpha (PPARα) and PPARγ co-activator-1 alpha and 1 beta in WAT resulting in resistance to HFD. The hyperinsulinemic euglycemic clamp studies showed that TRPM2 knockout mice are more insulin-sensitive and have elevated Akt and glycogen synthase kinase 3β phosphorylation in the heart, and reduced inflammation in the liver and adipose tissue [95]. These findings demonstrated that TRPM2 likely plays an important role in whole-body metabolism.

TRPM2, in addition to TRPM3, TRPM4, and TRPM5, was described in rodent insulinoma cell lines and in mouse islets. Moreover, transcripts of TRPM2, TRPM4 and TRPM5 have been identified in human islets [96,97], suggesting that they may have an important functional role in the regulation of pancreatic function and insulin secretion. TRPM2 is activated by a variety of stimuli including reactive oxygen species (e.g., H_2_O_2_), glucose, and incretins [62,98,99]. In TRPM2 knockout mice plasma insulin levels were reduced and glucose clearance was impaired. This was associated with decreased insulin secretion initiated by incretins and glucose, and was supported by the observation that in the β cells of TRPM2 knockout mice the increase of intracellular Ca^2+^ was impaired following stimulation with incretins or insulin [99]. Since TRPM2 activity can be modulated by adenine dinucleotides, intracellular Ca^2+^ levels, and incretin-induced PKA phosphorylation, the regulation of TRPM2 can be more complex; however, it is undoubtedly an important mechanism for insulin secretion [62]. Involvement of TRPM2 in type 1 and type 2 diabetes mellitus has been suggested and the current hypotheses, including impairment in insulin secretion due to dysfunction of TRPM2, have been discussed in detail previously [63].

TRPM3 is important for Zn^2+^ entry in β cells and might contribute to insulin synthesis [100]. This is supported by recent studies, which demonstrated expression of TRPM3 in rat pancreatic INS-1 insulinoma cells and in mouse pancreatic islets [101]. Previously it had been revealed that pregnenolone sulfate, a canonical TRPM3 agonist, induces Ca^2+^ influx. The increase of Ca^2+^ is due to activation of TRPM3 and l-type Ca^2+^ channels, and upregulation of genes, which lead to insulin secretion [64,65]. Recently, the roles of TRPM3 and l-type Ca^2+^ channels were clarified [101]. The authors concluded that both TRPM3 and l-type Ca^2+^ channels are necessary for the pregnenolone sulfate mediated gene stimulation in INS-1 cells cultured in low-glucose medium. These data suggest that activation of TRPM3 following pregnenolone sulfate stimulation leads to an initial Ca^2+^ influx followed by activation of l-type Ca^2+^ channels [65,101].

More recently, a synthetic small-molecule TRPM3 agonist, CIM0216, has been shown to stimulate insulin release [60]. Pregnenolone sulfate, the well-known agonist of TRPM3 and CIM0216, caused Ca^2+^ increase in pancreatic islets and a dose-dependent increase of insulin release. The increase of insulin release was not observed in TRPM3-deficient islets, further confirming the findings [60].

TRPM4 has been identified in several β cell lines suggesting non-species specific TRPM4 expression [102]. TRPM4 currents were characterized with a biphasic pattern following Ca^2+^ perfusion; however, the activation and inactivation time varied among cell lines. Inhibition of TRPM4 decreased the magnitude of Ca^2+^ signals, and it has been shown that blockade of TRPM4 decreased glucose stimulated insulin secretion in INS-1 cells [103]. Marigo and co-workers suggested that in β cells, TRPM4 may have a critical role in the regulation of membrane potential oscillations during glucose stimulation [102,103]. In contrast, TRPM4 knockout mice did not show differences in glucose tolerance test and glucose-induced insulin secretion from isolated islets compared to wild-type mice [104,105]. In addition, TRPM4 was proposed to be involved in glucagon secretion [105].

TRPM5 is important for the Ca^2+^ activated cation current in pancreatic β cells since this current was reduced in TRPM5 knockout mice [66]. TRPM5 is suggested to play crucial role during glucose stimulation, and it is likely responsible for the fast glucose-induced oscillations of membrane potential and Ca^2+^ [66]. These fast oscillations are more efficient to trigger insulin release [106]. Furthermore, impaired glucose tolerance was observed in TRPM5 knockout mice, supporting an important role for TRPM5 in the regulation of metabolism [66]. Its role in the control of metabolism is further demonstrated by the existence of TRPM5 in the enteroendocrine cells in the gastrointestinal tract. Specifically, TRPM5 is expressed in the GLP-1 secreting L-cells, which are important for controlling proper glucose homeostasis [69].

In addition, TRPM5 plays a role in taste signaling, and TRPM5 knockout mice develop severe impairment in sweet, bitter, and umami taste signaling [107,108,109]. Interestingly, members of taste signaling pathways including TRPM5 were identified in gastrointestinal L-cells, suggesting that sweet taste signaling is directly involved in the release of GLP-1, and thus can have an important role in glucose-mediated control of metabolism [69]. Manipulation of taste is also an approach of pharmacological interventions for obesity prevention, and TRPM5 has been investigated for potential taste modifications; details can be found elsewhere [110,111]. In addition, TRPM2, TRPM4, and TRPM5 channels are identified in the brain and may play a role in neuronal excitability, cell death, or neurodegeneration; however, their functional role in autonomic centers has not been described [19,75,76] (Table 1).

## 4. Implications for TRPA1

TRPA1, currently the only member of the ankyrin family, was named after the high number of ankyrin repeats. TRPA1 is a voltage-dependent, Ca^2+^-permeable cation channel. Like many of the TRP channels, TRPA1 is also modulated by herbal compounds, suggesting the potential for alternative dietary therapies. One of the natural compounds activating TRPA1 is cinnamon, a widely used spice, which originates from the bark of trees of the *Cinnamomum* genus. In one study, cinnamon treatment has been shown to improve the glucose and lipid profiles of type 2 diabetic patients [112], while other studies showed moderate improvement of glucose levels [113] or no effect [114]. We have to note that age, sex, length of the disease, and many other variables may play a role in the outcome of the human studies; therefore, it is too early to make a conclusion regarding the dietary benefits of cinnamon.

One of the main ingredients of cinnamon is cinnamaldehyde, which is a potent agonist of TRPA1. Cinnamaldehyde effect was associated with inhibition of ghrelin secretion and gastric emptying, whereas improved insulin sensitivity was observed [68]. In mice fed with high-fat high-sucrose diet, cinnamaldehyde ingestion was associated with reduced visceral adipose tissue [115] and increased fatty acid oxidation [68]. Recently it was shown that cinnamaldehyde (10 mg/kg) administration prevented the increase of weight gain caused by HFD [73]. Serum leptin levels and leptin/ghrelin ratio, a marker of weight gain, were decreased in the cinnamaldehyde-treated HFD groups. In addition, cinnamaldehyde treatment increased the expression levels of anorexigenic genes including pro-opiomelanocortin, urocortin, BDNF, and cholecystokinin [73]. The study also determined that cinnamaldehyde prevented visceral WAT accumulation, increased BAT activity and reduced inflammation, but did not affect gut microbial composition. Improved fasting blood glucose levels and glucose tolerance were observed in ob/ob mice following cinnamon extract treatment [116]. This was associated with improved insulin sensitivity, locomotor activity and improved brain activity.

Allyl isothiocyanate (AITC), an ingredient of mustard, horseradish, and wasabi, is also a potent TRPA1 agonist. It has been shown that intravenous injection of AITC induces adrenalin secretion. This response was attenuated in the presence of cholinergic blockers, suggesting activation of the adrenal sympathetic nerve through the central nervous system [117]. AITC increased thermogenesis and expression of UPC1 [74]. Recently, dietary AITC was reported to protect against free fatty acid induced insulin resistance, and it increased mitochondrial activity in skeletal muscle cells [118]. Dietary AITC reduced diet-induced obesity in C57Bl/6 mice and improved blood lipid profile compared to HFD-treated mice. AITC also reduced high fat induced hepatic steatosis and decreased hyperglycemia, hyperinsulinemia, HbA1C levels and ameliorated insulin resistance [118]. These findings suggest that activation of TRPA1 likely have beneficial effects; however, further studies are necessary to reveal the exact underlying mechanisms.

Multiple methodological approaches were used to reveal TRPA1 expression in rat pancreatic cells [61]. Expression of TRPA1 was confirmed in beta, but not in glucagon-secreting alpha cells, and activation of TRPA1 stimulated insulin release synergistically with ATP-dependent potassium channel (K_ATP_) blockade [61]. The latter is further supported with the findings that glibenclamide, a widely used K_ATP_ channel inhibitor is an agonist of TRPA1 [59], and it has been suggested that the synergistic effect of TRPA1 and K_ATP_ channels underlies the hyperinsulinism in patients with glibenclamide treatment.

Similar to TRPM5, TRPA1 is expressed in L-cells and TRPA1 agonist administration into the duodenum or by gavage increased GLP-1 secretion [67]. On the other hand, the effect was not eliminated in TRPA1 knockout mice. GLP-1 levels did not change following activation of TRPA1 despite elevation of peptide YY, and reduced gastric emptying and food intake [58]. In dogs, following AITC, gastric and jejunum motility was increased, and this effect was prevented with ruthenium red [119]. The potential role of TRPA1 on pancreatic, adipose tissue, and the autonomic nervous system and its importance as a dietary supplement has been recently reviewed [26].

## 5. Conclusions

TRP channels are expressed in many tissues and organs important for the maintenance of whole body metabolism. Results from dietary supplementation of TRP ligands (e.g., capsaicin) are controversial, either showing beneficial effects on body weight, metabolism, and hormone levels, or no effects. The target tissue of the dietary supplementation is not entirely clear since many tissues including the adipose tissue, the pancreas, and even the central nervous system could be modulated by the components of the diet. TRP channels have benefits; however, currently it is not clear whether activation or inhibition, central or peripheral mechanisms, diet or topical administration, or even which tissue/organ is the most critical. In summary, further research is needed before final conclusions are available, but undoubtedly TRP channels are good potential targets for weight and diabetes management.

## Figures and Tables

**Table 1:** Transient receptor potential (TRP) channels and their potential role in metabolism.

Title	TRPV1	TRPA1	TRPM2	TRPM3	TRPM5
Natural ligands for dietary interventions	capsaicin; red pepper; chili; capsinoids; capsiate	cinnamon; cinnamaldehyde; AITC			
Synthetic small molecule modulators	BCTC [56]; AZV1 [57]; AMG 517	methyl syringate [58]; glibenclamide [59]		CIM0216 [60]	
Pancreas	insulin + [40]	insulin + [59,61]	insulin + [62,63]	insulin + [60,64,65]	insulin + [66]
GI hormones and GI tract	GLP-1 + [47] ghrelin − [47]	GLP-1 + [67] ghrelin − [68]; peptide YY [58]; gastric and gut motility − [58,68]			GLP-1 + [69]
WAT	adipogenesis − [9]; browning + [12,55]; fat oxidation + [45,70]; leptin − [70,71]; adiponectin + [72]	fat oxidation + [68]; leptin − [73]			
BAT	thermogenesis + [12,45,46,71]	thermogenesis + [73,74]			
Nervous system	satiety + [48]; neuronal excitability + [21,22,42]; gene expression [71]	gene expression [73]	neuronal excitability [75]		neuronal excitability + [76]

AITC: allyl isothiocyanate; GLP-1: glucagon-like peptide 1; +: increase, −: decrease.
